# A combined association test for rare variants using family and case-control data

**DOI:** 10.1186/s12919-016-0033-x

**Published:** 2016-10-18

**Authors:** Peng-Lin Lin, Wei-Yun Tsai, Ren-Hua Chung

**Affiliations:** 1 Department of Medical Science, National Tsing Hua University, Hsin-Chu, Taiwan; 2 Division of Biostatistics and Bioinformatics, Institute of Population Health Sciences, National Health Research Institutes, Zhunan, Miaoli Taiwan

## Abstract

Statistical association tests for rare variants can be classified as the burden approach and the sequence kernel association test (SKAT) approach. The burden and SKAT approaches, originally developed for case–control analysis, have also been extended to family-based tests. In the presence of both case–control and family data for a study, joint analysis for the combined data set can increase the statistical power. We extended the Combined Association in the Presence of Linkage (CAPL) test, using both case–control and family data for testing common variants, to rare variant association analysis. The burden and SKAT algorithms were applied to the CAPL test. We used simulations to verify that the CAPL tests incorporating the burden and SKAT algorithms have correct type I error rates. Power studies suggested that both tests have adequate power to identify rare variants associated with the disease. We applied the tests to the Genetic Analysis Workshop 19 data set using the combined family and case–control data for hypertension. The analysis identified several candidate genes for hypertension.

## Background

Rare variants may contribute to a large portion of disease risks [1]. Rare variant association tests can be classified as the burden test [2] and the sequence kernel association test (SKAT) [3]. The burden test, assuming variants have the same direction of effects on a disease, collapses minor alleles at variants in a region and compares the difference in allele frequencies for the collapsed alleles between cases and controls. SKAT uses a regression framework and a variance-component test to consider variants with different directions of effects. The burden and SKAT approaches, originally developed for case–control analysis, have been extended to family-based tests [4, 5]. In the presence of both case–control and family data for a study, such as the Genetic Analysis Workshop 19 (GAW19) data sets, joint analysis for the combined data set can increase the statistical power. FamSKAT [6], which accounts for familial correlation based on kinship coefficients in a linear mixed model, may be able to use both family and unrelated samples. However, FamSKAT was developed for quantitative trait. Extending the model to dichotomous trait while properly considering family structures remains challenging [7]. We extended the Combined Association in the Presence of Linkage (CAPL) test [8] to rare variant analysis. The CAPL test uses both case–control and family data, and properly considers population stratification with a clustering algorithm. We applied the burden and SKAT algorithms to the CAPL test, subsequently referred to as the CAPL-burden and the CAPL-SKAT, respectively. We applied the tests to the GAW19 data set using the combined family and case–control data. We used the real trait values to define the hypertension status. Some candidate genes for hypertension were identified in the analysis.

## Methods

### The GAW19 data

The GAW19 data set consists of 20 large Mexican American families with a total of 959 individuals and 1944 unrelated individuals. The family data include 464 individuals for whom whole genome sequencing data are available, while the sequences for other family members were imputed based on the sequenced individuals. Admixture analysis for the family data suggested that most of the family ancestry is European and Native American, where the proportions of the two ancestries in each individual are different [9]. The data for the unrelated individuals were whole exome sequenced. We used the real trait values to analyze the odd chromosomes. For family data, individuals were affected if at least one of their hypertension diagnoses was hypertensive, while other individuals were unaffected. For case–control data, individuals with systolic blood pressure (SBP) 140 or greater, diastolic blood pressure (DBP) 90 or greater, or taking blood pressure medication were affected, while others were unaffected. Variants were annotated using SeattleSeq (http://snp.gs.washington.edu/SeattleSeqAnnotation138/). We performed gene-based tests by testing the association of all variants in exons within a gene with the disease.

### Quality control

We used the PLINK [10] PI_HAT statistic, which is the proportion of loci that are identity-by-descent (IBD) between a pair of individuals, to examine the relatedness among the 1944 unrelated individuals. We removed an individual if the median of PI_HAT of the individual with others was greater than 0.05, which is slightly below the kinship coefficient of first cousin (i.e., 0.0625). Although the CAPL test considers familial correlation in the test, family structures need to be specifically provided in the CAPL test. Therefore, individual pairs with PI_HAT between 0.15 and 0.70 were also removed. Variants with missing rates greater than 10 % in either the family or the unrelated data were removed. The family and unrelated data were merged with the union of variants in the two data sets. Variants with Hardy-Weinberg equilibrium test *p*-values less than 10^−4^ in the merged data were removed. As we focused on analyzing rare variants, variants with minor allele frequencies (MAFs) greater than 5 % were removed.

### The CAPL test

We first review the CAPL test statistic, which is the fundamental statistic in the proposed test. For a nuclear family *i*, let *X*_*i*_ be the number of a specific allele in affected siblings, *G*_*i*_ be the siblings’ genotypes, *A* be the siblings’ affection status, *GP*_*j*_ be the parental mating type, *N*_*ij*_ be the number of alleles in *GP*_*j*_, and *ψ* be the set of all possible parental mating types conditional on *G*_*i*_. The CAPL test statistic *T*_*i*_ is calculated as

1 $$ {T}_i={X}_i-{\displaystyle {\sum}_{j\in \psi }P\left(G{P}_j\left|{G}_i,A\right.\right){N}_{ij}} $$

When parental genotypes are available, *ψ* is the observed genotype. A case or a control is treated as a single offspring with two missing parents and *T*_*i*_ can be calculated. Assuming there are *m* populations in the data, *P*(*GP*|*G*, *A*) in equation (1) can be modified as

2 $$ P\left(GP\left|G,A\right.\right)={\displaystyle {\sum}_{m=1}^MP\left(GP, pop=m\left|G,A\right.\right)={\displaystyle {\sum}_{m=1}^MP\left(GP\left|G,A, pop=m\right.\right)}}P\left( pop=m\left|G,A\right.\right) $$

Calculating the probabilities in equations (1) and (2) involves the estimation of parameters for the parental mating types, the IBD status in affected siblings, and the probability that a family is in a given population. These parameters are estimated based on the expectation-maximization (EM) algorithm. The sum of *T*_*i*_ over all families is the CAPL statistic *T*. A bootstrap procedure is used to estimate the variance of *T* [11]. The CAPL statistic for a variant takes the form $\frac{T}{\sqrt{\widehat{Var}(T)}}$, which follows a standard normal distribution under the null hypothesis of no linkage or no association.

### The rare variant CAPL tests

We applied the burden approach based on the weighted-sum method [2] and SKAT [3] to the CAPL statistic. Assuming there are *N* nuclear families and *M* variants, the CAPL-burden test statistic is defined as *B* = ∑_*k* = 1_^*M*^ *w*_*k*_ *T*_*k*_, where *w*_*k*_ is the weight for variant *k* and *T*_*k*_ is the CAPL statistic at variant *k*. Following Madsen and Browning [2], the weight *w*_*k*_ is $1/\sqrt{n{q}_k\left(1-{q}_k\right)}$, where *n* is the number of unrelated individuals and *q*_*k*_ is the estimated MAF based on the unrelated individuals for variant *k*. The CAPL-SKAT statistic is defined as *S* = ∑_*k* = 1_^*M*^ *u*_*k*_^2^ *T*_*k*_^2^. Following Wu et al. [3], we used *Beta*(*q*_*k*_; 1, 25), the beta density function with parameters of (1, 25), as the weight function for *u*_*k*_. We used the bootstrap statistics to evaluate the significance for *B* and *S*. Assuming *L* bootstraps are performed, the bootstrap CAPL statistics under the null hypothesis at variant *k* is ${T_{k_j}}^{*}={T}_{k_j}-{M}_k$ at bootstrap replicate *j*, where ${T}_{k_j}$ is the original bootstrap statistic for *T*_*k*_ at bootstrap replicate *j* and ${M}_k=\left({\displaystyle {\sum}_{j=1}^L{T}_{k_j}}\right)/L$. The burden and SKAT bootstrap statistics under the null are calculated as ${B}_j^{*}={\displaystyle {\sum}_{k=1}^M{w}_k{T}_k{{}_{{}_j}}^{*}}$ and ${S}_j^{*}={{\displaystyle {\sum}_{k=1}^M{u_k}^2\left({T}_k{{}_{{}_j}}^{*}\right)}}^2$ at bootstrap replicate *j*. The *p*-values for *B* and *S* are calculated as (*number of B*_j_^*^ ≥ *B*)/*L* and (*number of S*_j_^*^ ≥ *S*)/*L*, respectively. In our analysis, *L* was set as 10,000.

### Simulation studies

As the simulated trait data for unrelated individuals were not available when we attempted to evaluate the statistical properties of the proposed tests, we used computer simulations to evaluate the type I error rates and power for the CAPL-burden and CAPL-SKAT. HAPGEN2 [12] was used to simulate 2 sets of haplotypes in the microtubule-associated protein 4 (*MAP4*) gene based on the 1000 Genomes project sequence data for the CEU (Utah residents with Northern and Western European ancestry) and MXL (Mexican ancestry in Los Angeles, California) populations, where each set consists of 10,000 haplotypes. SeqSIMLA [13] was used to simulate families and case–control data based on the haplotypes. A total of 50 nuclear families with 3 siblings, where at least one sibling was affected, and 300 cases and 1000 controls were simulated. The sample sizes were similar to the GAW19 data used in our data analysis. We performed admixture analyses using the AXMITURE software [14] for the CEU- and MXL-simulated samples, and observed similar global ancestry proportions for the MXL samples as the proportions in the GAW19 family data reported by Thornton et al. [9], where large proportions for the CEU samples were inferred from the same ancestry. We selected 50 and 100 rare variants with MAFs of less than 5 % for the tests. The disease prevalence was 5 %. The type I error rates were calculated based on 5000 replicates of the simulated data. For the power simulations, we randomly selected 10 variants with MAFs of less than 1 % as the disease loci. We assumed that the population attributable risk (PAR) was 1 % for each disease locus, and similar to that of Madsen and Browning [2], the odds ratio (OR) for disease locus *i* was calculated as *OR*_*i*_ = 1 + (0.01/(0.99 × *MAF*_*i*_)), where *MAF*_*i*_ is the MAF for *i*. We also simulated the scenario where 50 % of the disease loci were protective. The OR for protective variant *i* was specified as 1/*OR*_*i*_. The power was calculated based on 1000 replicates of the simulated data.

## Results

### Simulation studies

Table 1 shows the type I error rates and the 95 % confidence intervals for the CAPL-burden and the CAPL-SKAT with different numbers of tested variants in the 2 populations. As seen in the results, the type I error rates for the 2 tests were properly maintained at the 0.05 significance level under different scenarios. Similar results were obtained at the 0.01 significance level (data not shown). Most of the confidence intervals include the expected levels. Table 2 shows the power comparison between the CAPL-burden and the CAPL-SKAT under 4 scenarios (Scen1 to Scen4) for MXL. When all of the causal variants had risk effects, the CAPL-burden had similar power with the CAPL-SKAT for both 50 and 100 variants being tested. However, when 50 % of the causal variants were changed to be protective, the CAPL-burden had a significant power loss while the CAPL-SKAT still maintained power. This is as expected as the CAPL-SKAT accounts for the directions of effects in the test. We also combined the CEU and MXL simulated replicates for Scen3, and the power for the 2 tests is close to 1, suggesting that the tests maintained power in highly stratified samples.

**Table 1:** Type I error rates and 95 % confidence intervals for the CAPL-burden and CAPL-SKAT at α = 0.05

Population	Number of variants	CAPL-burden	CAPL-SKAT
CEU	50	0.056 (0.049, 0.062)	0.047 (0.041, 0.052)
CEU	100	0.049 (0.043, 0.054)	0.042 (0.036, 0.047)
MXL	50	0.050 (0.043, 0.056)	0.045 (0.039, 0.050)
MXL	100	0.049 (0.043, 0.054)	0.054 (0.047, 0.060)
CEU + MXL	100	0.045 (0.039, 0.051)	0.043 (0.037, 0.048)

**Table 2:** Power comparison between the CAPL-burden and the CAPL-SKAT for the MXL population

Scenario	Number of variants	% of Protective variants	CAPL-burden	CAPL-SKAT
Scen1	50	0	0.983	0.953
Scen2	50	50	0.211	0.850
Scen3	100	0	0.874	0.898
Scen4	100	50	0.097	0.721

### The rare variant analysis for hypertension

Because the CAPL can only analyze nuclear families, we split the extended pedigrees to non-overlapping nuclear families. A total of 863 individuals in the 1944 unrelated samples were removed because of their cryptic relatedness based on the PI_HAT statistics. In the combined data, there were 1509 individuals, including 948 unrelated controls and 305 unrelated cases, and 256 individuals in 47 nuclear families. There were 2,649,583 variants after quality control. A total of 12,340 genes were analyzed. In the CAPL, we specified the number of populations as 2 for the clustering algorithm, where the family data and unrelated data were clustered in two populations, to account for the batch effect for the 2 data sets.

Table 3 lists the top 10 significant genes. Although none of the tests for the top 10 genes passed the multiple testing correction threshold (i.e., 0.05/12340 = 2.34 × 10^− 6^), some genes, which are underlined in Table 3, have functional implications for hypertension. Among the underlined genes, G protein-coupled receptor, class C, group 5, member C (GPRC5C) is particular interesting. GPRC5C may have cellular effect between retinoic acid and the G-protein-coupled receptor (GPCR) signal transduction pathway [15]. Dysfunction of the GPCR signal transduction in the cardiovascular system may increase the risk of hypertension [16].

**Table 3:** The 10 most significant genes for hypertension

Gene^a^	*p* Value	Test	Gene^a^	*p* Value	Test
*KRTAP21-2*	1.00E-04	CAPL-SKAT	*UBE2Q2*	5.00E-04	CAPL-SKAT
*GPRC5C*	1.29E-04	CAPL-burden	*TVP23C-CDRT4*	7.00E-04	CAPL-SKAT
*TAS2R38*	2.00E-04	CAPL-SKAT	*ELMO1*	7.00E-04	CAPL-SKAT
*CCND1*	4.00E-04	CAPL-SKAT	*EXT2*	8.00E-04	CAPL-burden
*ANXA2*	5.00E-04	CAPL-SKAT	*CYFIP1*	1.00E-03	CAPL-burden

^a^Genes with functional implications for hypertension are underlined

Another underlined gene, ubiquitin-conjugating enzyme E2Q family member 2 (*UBE2Q2*), has been identified to have association with chronic kidney disease [17]. Chronic kidney disease can induce several cardiovascular diseases, including hypertension [18]. The last underlined gene, engulfment and cell motility 1 (*ELMO1*), is a susceptible gene in diabetic nephropathy [19], and hypertension is highly prevalent in diabetic nephropathy patients [20].

## Discussion and conclusions

We extended the CAPL test to rare variant association tests. The significance for the CAPL-burden and the CAPL-SKAT statistics are assessed with their bootstrap statistics under the null hypothesis. Among the 10 most significant genes for hypertension, we identified several candidate genes for hypertension. More research is needed to study the role of the candidate genes in hypertension.

For the power studies, we also evaluated the performance of the proposed tests for testing variants obtained based on MAF thresholds of 10 %, 20 %, 30 %, 40 %, and 50 % for Scen3 (data not shown). The results suggest that the CAPL-SKAT had slight power loss with the increase of the MAF thresholds, whereas the power for the CAPL-burden decreased to 0.36 when the proportion of causal variants in the variants being tested decreased to 7 % at the MAF threshold of 40 %. Consequently, in practice, the CAPL-SKAT should be performed if common variants are included in the analysis.

The CAPL uses a clustering algorithm, which assumes family members have the same genetic background across the genome, to identify subpopulations. Our simulation results showed that the CAPL test maintained correct type I error rates for the admixed population, where the assumption of homogeneous genetic background across the genome in all family members is violated. It is possible to extend the CAPL test to account for population admixture by calculating *P*(*pop* = *m*|*G*, *A*) in equation (2) based on the local admixture probabilities estimated using software such as LAMP-LD or LAMP-HAP [21]. Correlation in genotypes for siblings needs to be considered when calculating the local admixture probabilities. As LAMP-LD assumes independent samples and LAMP-HAP uses only trio information, it will be of interest to evaluate the robustness of the CAPL-burden and CAPL-SKAT tests when the local admixture probabilities estimated from the software are incorporated in the methods.

It is possible to combine the CAPL-burden and CAPL-SKAT into a test, using algorithms such as SKAT-O, which can reduce the number of tests. Moreover, the CAPL-burden and CAPL-SKAT currently focus on analyzing rare variants. It is possible to extend the tests to accommodate both common and rare variants, using algorithms such as those found in Chung et al. [22] and Saad and Wijsman [23]. As more sequencing studies are performed on either the family or the case–control designs, the CAPL-burden and CAPL-SKAT will be useful for identifying candidate genes using the combined case–control and family data.
